# Estimating the phylogeny of geoemydid turtles (Cryptodira) from landmark data: an assessment of different methods

**DOI:** 10.7717/peerj.7476

**Published:** 2019-08-22

**Authors:** Eduardo Ascarrunz, Julien Claude, Walter G. Joyce

**Affiliations:** 1 Department of Geosciences, University of Fribourg, Fribourg, Switzerland; 2 Institut des Sciences de l’Évolution de Montpellier, UMR UM/CNRS/IRD/EPHE, Montpellier, France

**Keywords:** Geometric morphometrics, Turtles, Phylogenetics, Landmark analysis, Systematics

## Abstract

**Background:**

In the last 20 years, a general picture of the evolutionary relationships between geoemydid turtles (ca. 70 species distributed over the Northern hemisphere) has emerged from the analysis of molecular data. However, there is a paucity of good traditional morphological characters that correlate with the phylogeny, which are essential for the robust integration of fossil and molecular data. Part of this problem might be due to intrinsic limitations of traditional discrete characters. Here, we explore the use of continuous data in the form of 3D coordinates of homologous landmarks on the turtle shell for phylogenetic inference and the phylogenetic placement of single species on a scaffold molecular tree. We focus on the performance yielded by sampling the carapace and/or plastral lobes and using various phylogenetic methods.

**Methods:**

We digitised the landmark coordinates of the carapace and plastron of 42 and 46 extant geoemydid species, respectively. The configurations were superimposed and we estimated the phylogenetic tree of geoemydids with landmark analysis under parsimony, traditional Farris parsimony, unweighted squared-change parsimony, maximum likelihood with a Brownian motion model, and neighbour-joining on a matrix of pairwise Procrustes distances. We assessed the performance of those analyses by comparing the trees against a reference phylogeny obtained from seven molecular markers. For comparisons between trees we used difference measures based on quartets and splits. We used the same reference tree to evaluate phylogenetic placement performance by a leave-one-out validation procedure.

**Results:**

Whatever method we used, similarity to the reference phylogeny was low. The carapace alone gave slightly better results than the plastron or the complete shell. Assessment of the potential for placement of single species on the reference tree with landmark data gave much better results, with similar accuracy and higher precision compared to the performance of discrete characters with parsimony.

## Introduction

Geometric morphometrics by means of landmark analysis have had considerable success in the description of subtle or complex shape variation that is otherwise difficult to characterise (Adams, Rohlf & Slice, 2013). Thus, landmark data would seem an attractive source of information for phylogenetic inference in cases where the definition of discrete morphological characters is challenging. However, the use of these data has been historically limited. Several methods have been proposed for the phylogenetic analysis of landmark data (Zelditch, Fink & Swiderski, 1995; Caumul & Polly, 2005; González-José et al., 2008; Klingenberg & Gidaszewski, 2010), but for the most part they have been controversial (Rohlf, 1998; Zelditch et al., 2004; Adams et al., 2011) and failed to gain significant acceptance.

The most recent development within the framework of maximum parsimony is the work of Catalano and Goloboff, who extended the reasoning and algorithms of Farris optimisation to two and three-dimensional space (Catalano, Goloboff & Giannini, 2010; Goloboff & Catalano, 2011; Catalano & Goloboff, 2012), allowing for the application of the criterion of maximum parsimony to the superposition of landmark coordinates and full topological searches. Following Perrard, Lopez-Osorio & Carpenter (2016), we dub this approach landmark analysis under parsimony (LAUP). Much earlier, the work of Felsenstein (1973, 1981) provided a computationally tractable way for calculating the likelihood on continuous characters under Brownian motion models, but its application had received only peripheral attention until the recent work of Parins-Fukuchi (2017, 2018). Here, we present a case study of the application of LAUP, maximum likelihood, and other methods in resolving the phylogeny of geoemydid turtles from shell characters.

Geoemydidae is a large clade comprising around 20% of the currently recognised turtle species (Rhodin et al., 2017). In the last 20 years, a broad consensus of the evolutionary history of the group has emerged from molecular phylogenetic analyses (Barth et al., 2004; Feldman & Parham, 2004; Spinks et al., 2004, 2012; Diesmos et al., 2005; Sasaki et al., 2006; Le, McCord & Iverson, 2007; Le & McCord, 2008; Guillon et al., 2012). In contrast, phylogenetic analyses based on morphological characters have yielded very inconsistent results (Garbin, Ascarrunz & Joyce, 2018), and, consequently, the efforts to determine the relationships of fossil specimens have been limited in various ways. For instance, several palaeontological studies restricted the scope of their analyses to small subclades (Joyce & Lyson, 2010; Naksri et al., 2013; Joyce et al., 2013), while others did not attempt to use any algorithmic methods (Das, 1997; Claude & Tong, 2004; Hirayama, Kaneko & Okazaki, 2007; Claude, Suteethorn & Tong, 2007; Claude et al., 2012; Takahashi et al., 2013). Meanwhile, the most ambitious revisions of the systematic status of fossil geoemydid material (Hervet, 2004, 2006) relied on analyses that did not include enough extant species to adequately assess global phylogenetic relationships and potential character conflict.

Major problems with morphological data seem to be the presence of extensive polymorphism and homoplasy, and the scarcity of discrete characters in the geoemydid shell (geoemydid skulls are scant in the fossil record, and appendicular elements are generally deemed to be mostly uninformative). The use of landmarks is therefore an interesting alternative to traditional discrete characters for at least two major reasons in this clade:

1. Most of the traditional shell characters are positional, generally referring to the position of a suture or sulcus relative to another. This kind of phenotypic variation is continuous by nature. Being able to represent and analyse these changes as continuous allows researchers to obtain a more fine-grained resolution from the same character and avoids the problem of arbitrarily binning continuous variation into discrete states.
2. Landmarks allow capturing and representing some shell variation that may be apparent to researchers, but difficult to characterise and to code into states. Examples of this variation include the degree of ‘doming’ and the contour of the carapace or the degree of roundness of the plastral lobes.

Thus, the use of landmarks could help palliate the problem of the scarcity of discrete states. Geometric morphometric methods have been applied to geoemydids in studies of diversification, morphological convergence, and adaptation (Claude et al., 2003, 2004; McLaughlin & Stayton, 2016) and shape asymmetry (Rivera & Claude, 2008), and also to validate character definitions (Russell & Jamniczky, 2004). To our knowledge, the present study is the first attempt to use landmark data to reconstruct the phylogeny of geoemydids, or any other clade of turtles.

We have two main objectives in our assessment of the performance of various phylogenetic reconstruction methods with our dataset. The first is to provide one more study of the performance of phylogenetic reconstruction with empirical morphometric data, which will contribute to understanding the behaviour and usefulness of the methods for future applications. The second is to assess the potential usefulness of morphometric data for determining the phylogenetic position of geoemydid species that are only known from fossil remains. With this second goal in mind, we had particular interest in the results of the analyses of plastron landmarks. Because turtle fossils are typically deformed and disarticulated by dorsoventral compression, fossil carapaces will seldom be adequate for analysis of their geometrical properties. In contrast, the original shape of plastra is mostly flat and will therefore be less affected by dorsoventral compression.

### Landmark analysis under parsimony

Several methods can be applied to morphometric data for estimating phylogenetic relationships (e.g. neighbour joining (NJ), linear parsimony, squared-change parsimony (SCP), maximum likelihood (ML)). Thanks to the nature of shape data, these methods can be applied in the shape space to reduce the length of the tree. While NJ works directly on distances without explicitly reconstructing ancestral character states, the three other methods estimate ancestral shapes from landmark data in order to minimise the morphological evolution of the tree according to an optimisation logic. LAUP differs from these methods because it considers individual landmarks as a unit for the optimisation procedure and introduces several new concepts to parsimony analysis. We will provide a brief account of the method to facilitate the comprehension of this paper, but interested readers are referred to the original publications (Catalano, Goloboff & Giannini, 2010; Goloboff & Catalano, 2011, 2016; Catalano & Goloboff, 2012). Catalano and Goloboff’s approach operates directly on landmark coordinates and attempts to minimise individual landmark displacements between ancestors and descendants along the tree, keeping evolutionary changes localised. This is achieved by estimating ancestral configurations at the internal nodes in a manner analogous to the estimation of character states in traditional parsimony. The landmark displacements are minimised as Euclidian distances, not squared-changes as in SCP.

The optimisation of the ancestral configurations is ‘spatial’ as opposed to ‘linear’ in the sense that the coordinates of a landmark are optimised as a multivariate character and not as three (or two) independent characters. However, the method of optimisation is not exact. Goloboff & Catalano (2011) implemented algorithms to find the ancestral position of a landmark by trying out a limited set of positions determined by a grid encompassing the range of the observed positions of the landmarks in the descendants. The ancestral position approximation can be refined by increasing the number of subdivisions of the grid or by repeating the procedure using a smaller grid around the position of the first approximation (‘grid nesting’). For other possible improvements, see Goloboff & Catalano (2011).

Another major aspect of LAUP is concerned with tree search and the landmark superposition (or ‘alignment’). Although it is possible to look for most parsimonious trees (MPTs) using the approach just described on any kind of superposition, including Procrustes, Catalano & Goloboff (2012) argue that it is more consistent with the logic of LAUP to superimpose the configurations to minimise the parsimony score of a given tree. Thus, it is possible to dynamically optimise both tree and alignment during tree search: an initial superposition is used to make a first estimation of tree topology, and then the superposition is adjusted by rotating the configurations to minimise the score of the estimated topology. The process is repeated until no further changes in topology and superposition are produced.

## Methods

### Reference phylogeny

We constructed a phylogeny of geoemydids from molecular data to use as a reference against which the analyses of morphometric data could be compared. We collected DNA sequences of 64 geoemydid species and two testudinid outgroups from seven loci: 12s, C-MOS, COI-5p, CYTB, R35, RAG1, and RAG2. COI-5p sequences were obtained from DNA barcode sequences available on the BOLD workbench (Ratnasingham & Hebert, 2007). Sequences of the other loci were collected from GenBank. We only selected sequences that were used in published studies. Our least inclusive analytical unit is the species. In some cases, we used sequences from different subspecies in order to maximise locus sampling per species.

We aligned all sequences with MUSCLE v.3.8.31 (Edgar, 2004), and performed manual corrections as necessary (Dataset S1). Selection of partition scheme and transition models was performed using the greedy merging strategy (Lanfear et al., 2014) implemented in IQ-TREE version 1.5.3 (multi-thread) (Nguyen et al., 2015). For the ML phylogenetic estimation, we ran 15 searches on IQ-TREE with an edge-proportional partition model (Chernomor, Von Haeseler & Minh, 2016) and the default search heuristic settings. Branch support was assessed with ultra-fast bootstrapping (Minh, Nguyen & Von Haeseler, 2013).

Finally, the resulting ML tree was adapted to match the species sampling of each morphometric dataset (see below) by dropping superfluous species and replacing the testudinid outgroups by the emydid *Malaclemys terrapin* for the reasons explained in the following section.

### Specimen and landmark sampling

We examined specimens from collections in the following institutions in the USA: the Field Museum of Natural History (FMNH) in Chicago, Illinois, the Chelonian Research Institute (PCHP) in Oviedo, Florida, the Museum of Comparative Zoology (MCZ) in Cambridge, Massachusetts, and the Yale Peabody Museum (YPM) in New Haven, Connecticut. We restricted our sampling to specimens with visible bony sutures and without supernumerary bone plates or scutes. The full details of all the specimens examined is given in Dataset S2. In total, 103 carapaces and 98 plastra were used in this study (Table S1), all of adult specimens, representing 47 out of the 71 currently recognised geoemydid species. On average, each species is represented by 2.3 carapace specimens and 2.1 plastron specimens (Table S2). Our sample is not sufficient to cover all subspecies as well as possible sexual dimorphism and ontogenetic variation within geoemydid species, in part because skeletal material is rare and typically lacks sex information. These are clear limitations in our data, as uneven sampling of sexual and size variation can feasibly lead to artefactual clustering of species (e.g. species represented only by females). We note, however, that these limitations are inherent in palaeontological material, and are more likely to affect the resolution of species that are closely related than to cause major errors in the estimation of the global topology.

Geoemydids and testudinids (clade Testuguria) are more closely related to each other than to emydids (Spinks et al., 2004). However, taking into account extant species and the fossil record, it is apparent that the shell morphology of testudinids is highly autapomorphic, with features such as high doming, alternating patterns of costal width, and very long anterior gular projections for most species. We consider, in contrast, that the shell of emydids is more conservative and morphologically closer to the shape of ancestral testugurians. Therefore, we selected the emydid *M. terrapin* to be the outgroup for our analyses.

**Figure 1:**
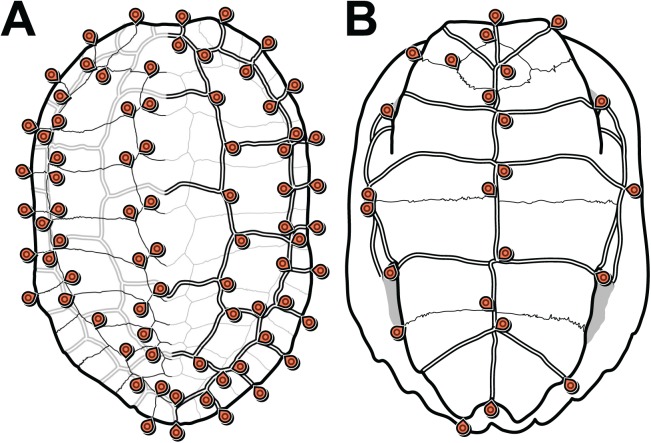
Carapace (A) and plastron (B) landmarks used in this study. The left side of each illustration (anatomical left of the carapace, anatomical right of the plastron) shows landmarks on bone sutures. The right side shows landmarks on the scute sulci. Suture and sulcus landmarks were acquired from both sides of each specimen. Thin lines represent bone sutures. Thick double lines represent scute sulci.

We defined 152 landmarks for the carapace (Table 1; Dataset S3) and 48 (Table 2; Dataset S4) landmarks for the plastron (Fig. 1). Most of these landmarks (150 and 46, respectively) are laterally symmetric, i.e. they have a homologous counterpart on the opposite side of the shell. Because of this redundancy, we reduced the number of effective landmarks for the phylogenetic analyses to 77 landmarks on the carapace and 25 on the plastron (see Data filtering and Procrustes superimposition).

**Table 1:** Carapace landmark definitions.

Landmark number	Side	Definition
1	Right	Nuchal, external contact with peripheral 1
2	Right	Nuchal, contact with peripheral 1 and costal 1
3	Right	Neural 1, contact with nuchal
4	Right	Neural 2, contact with neural 1
5	Right	Neural 3, contact with neural 2
6	Right	Neural 4, contact with neural 3
7	Right	Neural 5, contact with neural 4
8	Right	Neural 5, contact with neural 6
9	Right	Posterior suprapygal, contact with posterior costal and peripheral series
10	Right	Posterior suprapygal, contact with pygal
11	Right	Pygal, external contact with posterior peripheral
12	Right	Costal 1, internal contact with costal 2
13	Right	Costal 2, internal contact with costal 3
14	Right	Costal 3, internal contact with costal 4
15	Right	Costal 4, internal contact with costal 5
16	Right	Costal 5, internal contact with costal 6
17	Right	Costal 6, internal contact with costal 7
18	Right	Costal 7, internal contact with costal 8
19	Right	Costal 1, external contact with costal 2
20	Right	Costal 2, external contact with costal 3
21	Right	Costal 3, external contact with costal 4
22	Right	Costal 4, external contact with costal 5
23	Right	Costal 5, external contact with costal 6
24	Right	Costal 6, external contact with costal 7
25	Right	Costal 7, external contact with costal 8
26	Right	Peripheral 1, internal contact with peripheral 2
27	Right	Peripheral 2, internal contact with peripheral 3
28	Right	Peripheral 3, internal contact with peripheral 4
29	Right	Peripheral 4, internal contact with peripheral 5
30	Right	Peripheral 5, internal contact with peripheral 6
31	Right	Peripheral 6, internal contact with peripheral 7
32	Right	Peripheral 7, internal contact with peripheral 8
33	Right	Peripheral 1, external contact with peripheral 2
34	Right	Peripheral 2, external contact with peripheral 3
35	Right	Peripheral 3, external contact with peripheral 4
36	Right	Peripheral 4, external contact with peripheral 5
37	Right	Peripheral 5, external contact with peripheral 6
38	Right	Peripheral 6, external contact with peripheral 7
39	Right	Peripheral 7, external contact with peripheral 8
40	Left	Nuchal, external contact with peripheral 1
41	Left	Nuchal, contact with peripheral 1 and costal 1
42	Left	Neural 1, contact with nuchal
43	Left	Neural 2, contact with neural 1
44	Left	Neural 3, contact with neural 2
45	Left	Neural 4, contact with neural 3
46	Left	Neural 5, contact with neural 4
47	Left	Neural 5, contact with neural 6
48	Left	Posterior suprapygal, contact with posterior costal and peripheral series
49	Left	Posterior suprapygal, contact with pygal
50	Left	Pygal, external contact with posterior peripheral
51	Left	Costal 1, internal contact with costal 2
52	Left	Costal 2, internal contact with costal 3
53	Left	Costal 3, internal contact with costal 4
54	Left	Costal 4, internal contact with costal 5
55	Left	Costal 5, internal contact with costal 6
56	Left	Costal 6, internal contact with costal 7
57	Left	Costal 7, internal contact with costal 8
58	Left	Costal 1, external contact with costal 2
59	Left	Costal 2, external contact with costal 3
60	Left	Costal 3, external contact with costal 4
61	Left	Costal 4, external contact with costal 5
62	Left	Costal 5, external contact with costal 6
63	Left	Costal 6, external contact with costal 7
64	Left	Costal 7, external contact with costal 8
65	Left	Peripheral 1, internal contact with peripheral 2
66	Left	Peripheral 2, internal contact with peripheral 3
67	Left	Peripheral 3, internal contact with peripheral 4
68	Left	Peripheral 4, internal contact with peripheral 5
69	Left	Peripheral 5, internal contact with peripheral 6
70	Left	Peripheral 6, internal contact with peripheral 7
71	Left	Peripheral 7, internal contact with peripheral 8
72	Left	Peripheral 1, external contact with peripheral 2
73	Left	Peripheral 2, external contact with peripheral 3
74	Left	Peripheral 3, external contact with peripheral 4
75	Left	Peripheral 4, external contact with peripheral 5
76	Left	Peripheral 5, external contact with peripheral 6
77	Left	Peripheral 6, external contact with peripheral 7
78	Left	Peripheral 7, external contact with peripheral 8
79	Right	Cervical, external contact with marginal 1
80	Right	Cervical, contact with vertebral 1
81	Right	Vertebral 1, contact with vertebral 2
82	Right	Vertebral 2, contact with vertebral 3
83	Right	Vertebral 3, contact with vertebral 4
84	Right	Vertebral 4, contact with vertebral 5
85	Right	Vertebral 5, contact with marginals 11 and 12
86	Right	Pleural 1, contact with vertebral 1 and marginal 1
87	Right	Pleural 1, internal contact with pleural 2
88	Right	Pleural 2, internal contact with pleural 3
89	Right	Pleural 3, internal contact with pleural 4
90	Right	Pleural 4, contact with vertebral 5 and marginals
91	Right	Pleural 1, external contact with pleural 2
92	Right	Pleural 2, external contact with pleural 3
93	Right	Pleural 3, external contact with pleural 4
94	Right	Marginal 1, internal contact with marginal 2
95	Right	Marginal 2, internal contact with marginal 3
96	Right	Marginal 3, internal contact with marginal 4
97	Right	Marginal 4, internal contact with marginal 5
98	Right	Marginal 5, internal contact with marginal 6
99	Right	Marginal 6, internal contact with marginal 7
100	Right	Marginal 7, internal contact with marginal 8
101	Right	Marginal 8, internal contact with marginal 9
102	Right	Marginal 9, internal contact with marginal 10
103	Right	Marginal 10, internal contact with marginal 11
104	Right	Marginal 1, external contact with marginal 2
105	Right	Marginal 2, external contact with marginal 3
106	Right	Marginal 3, external contact with marginal 4
107	Right	Marginal 4, external contact with marginal 5
108	Right	Marginal 5, external contact with marginal 6
109	Right	Marginal 6, external contact with marginal 7
110	Right	Marginal 7, external contact with marginal 8
111	Right	Marginal 8, external contact with marginal 9
112	Right	Marginal 9, external contact with marginal 10
113	Right	Marginal 10, external contact with marginal 11
114	Right	Marginal 11, external contact with marginal 12
115	Left	Cervical, external contact with marginal 1
116	Left	Cervical, contact with vertebral 1
117	Left	Vertebral 1, contact with vertebral 2
118	Left	Vertebral 2, contact with vertebral 3
119	Left	Vertebral 3, contact with vertebral 4
120	Left	Vertebral 4, contact with vertebral 5
121	Left	Vertebral 5, contact with marginals 11 and 12
122	Left	Pleural 1, contact with vertebral 1 and marginal 1
123	Left	Pleural 1, internal contact with pleural 2
124	Left	Pleural 2, internal contact with pleural 3
125	Left	Pleural 3, internal contact with pleural 4
126	Left	Pleural 4, contact with vertebral 5 and marginals
127	Left	Pleural 1, external contact with pleural 2
128	Left	Pleural 2, external contact with pleural 3
129	Left	Pleural 3, external contact with pleural 4
130	Left	Marginal 1, internal contact with marginal 2
131	Left	Marginal 2, internal contact with marginal 3
132	Left	Marginal 3, internal contact with marginal 4
133	Left	Marginal 4, internal contact with marginal 5
134	Left	Marginal 5, internal contact with marginal 6
135	Left	Marginal 6, internal contact with marginal 7
136	Left	Marginal 7, internal contact with marginal 8
137	Left	Marginal 8, internal contact with marginal 9
138	Left	Marginal 9, internal contact with marginal 10
139	Left	Marginal 10, internal contact with marginal 11
140	Left	Marginal 1, external contact with marginal 2
141	Left	Marginal 2, external contact with marginal 3
142	Left	Marginal 3, external contact with marginal 4
143	Left	Marginal 4, external contact with marginal 5
144	Left	Marginal 5, external contact with marginal 6
145	Left	Marginal 6, external contact with marginal 7
146	Left	Marginal 7, external contact with marginal 8
147	Left	Marginal 8, external contact with marginal 9
148	Left	Marginal 9, external contact with marginal 10
149	Left	Marginal 10, external contact with marginal 11
150	Left	Marginal 11, external contact with marginal 12
151	Medial	Left and right marginal 12, medial contact between each other
152	Medial	Left and right marginal 12, distal contact between each other

**Table 2:** Plastron landmark definitions.

Landmark number	Side	Plastral lobe	Definition
1	Medial	Anterior	Epiplastra, external contact between each other
2	Medial	Posterior	Xiphiplastra, external contact between each other
3	Right	Anterior	Epiplastron, medial contact with entoplastron
4	Right	Anterior	Entoplastron, medial contact with hyoplastron
5	Right	Anterior	Hyoplastron, medial contact with hypoplastron
6	Right	Posterior	Hyoplastron, medial contact with hypoplastron
7	Right	Posterior	Hypoplastron, medial contact with xiphiplastron
8	Right	Anterior	Entoplastron, contact with epiplastron and hyoplastron
9	Right	Anterior	Epiplastron, lateral contact with hyoplastron
10	Right	Anterior	Axillary notch, inflection point
11	Right	Anterior	Hyoplastron, lateral contact with hypoplastron
12	Right	Posterior	Hyoplastron, lateral contact with hypoplastron
13	Right	Posterior	Inguinal notch, inflection point
14	Right	Posterior	Hypoplastron, lateral contact with xiphiplastron
15	Right	Posterior	Xiphiplastron, inflection point of external border
16	Left	Anterior	Epiplastron, medial contact with entoplastron
17	Left	Anterior	Entoplastron, medial contact with hyoplastron
18	Left	Anterior	Hyoplastron, medial contact with hypoplastron
19	Left	Posterior	Hyoplastron, medial contact with hypoplastron
20	Left	Posterior	Hypoplastron, medial contact with xiphiplastron
21	Left	Anterior	Entoplastron, contact with epiplastron and hyoplastron
22	Left	Anterior	Epiplastron, lateral contact with hyoplastron
23	Left	Anterior	Axillary notch, inflection point
24	Left	Anterior	Hyoplastron, lateral contact with hypoplastron
25	Left	Posterior	Hyoplastron, lateral contact with hypoplastron
26	Left	Posterior	Inguinal notch, inflection point
27	Left	Posterior	Hypoplastron, lateral contact with xiphiplastron
28	Left	Posterior	Xiphiplastron, inflection point of external border
29	Right	Anterior	Gular, medial contact with humeral
30	Right	Anterior	Humeral, medial contact with pectoral
31	Right	Anterior	Pectoral, medial contact with abdominal
32	Right	Posterior	Abdominal, medial contact with femoral
33	Right	Posterior	Femoral, medial contact with anal
34	Right	Anterior	Gular, lateral contact with humeral
35	Right	Anterior	Humeral, lateral contact with pectoral
36	Right	Anterior	Pectoral, lateral contact with abdominal
37	Right	Posterior	Abdominal, lateral contact with femoral
38	Right	Posterior	Femoral, lateral contact with anal
39	Left	Anterior	Gular, medial contact with humeral
40	Left	Anterior	Humeral, medial contact with pectoral
41	Left	Anterior	Pectoral, medial contact with abdominal
42	Left	Posterior	Abdominal, medial contact with femoral
43	Left	Posterior	Femoral, medial contact with anal
44	Left	Anterior	Gular, lateral contact with humeral
45	Left	Anterior	Humeral, lateral contact with pectoral
46	Left	Anterior	Pectoral, lateral contact with abdominal
47	Left	Posterior	Abdominal, lateral contact with femoral
48	Left	Posterior	Femoral, lateral contact with anal
49*	Right	–	Peripheral 3, external contact with peripheral 4
50*	Right	–	Peripheral 7, external contact with peripheral 8
51*	Left	–	Peripheral 3, external contact with peripheral 4
52*	Left	–	Peripheral 7, external contact with peripheral 8

**Note:**

The landmarks marked with asterisks (49–52) were not included in the analyses for this study. We show them in this list because they are present in the raw data we provide. These landmarks actually belong to the carapace, and were acquired together with the plastron landmarks in order to make it possible to realign the plastra with their respective carapaces. That alignment was not done in this study, because it’s not applicable to species with mobile plastra and to collection specimens in which the carapace and the plastron were preserved separately.

Our landmark definition scheme corresponds mostly to the landmarks of Claude et al. (2003), but we added landmarks for neural bones 1–5 (the number of neural bones in the posterior half of the carapace is often variable within a species), the marginal series, and the anterior peripheral series. We also adapted the definitions of the plastral landmarks to preserve all information related to right/left variation in our raw data, although side variation was not addressed in the present study.

Landmark coordinates were acquired in three dimensions with a Microscribe G digitising arm (Revware Inc., Raleigh, NC, USA) by E.A. The raw landmark data is given in Dataset S5. Each carapace and plastron was digitised twice to account for intra-operator error.

### Specimen identification

For specimen identification, we relied primarily on collection labels and catalogue information, but we verified those identifications against morphological characters and current information on geographic ranges when it was possible. Specimens for which the species attribution seemed dubious were discarded for the construction of the datasets for the analyses.

### Data filtering and Procrustes superposition

We excluded right/left variation and developmental abnormalities from the data. For this, we first filtered our data to exclude specific landmarks from regions of 10 carapace specimens with obvious developmental abnormalities, as inferred from extreme right/left asymmetry and by comparison to conspecifics. No plastra had abnormalities requiring this treatment. Then, we performed generalised procrustes analyses (GPA) and generated a consensus of the two digitisation replicates of each specimen. The specimen consensus of carapaces and plastra were symmetrised estimating the missing landmarks by reflection using the R package StereoMorph v.1.5.1 (Olsen & Westneat, 2015). As side variation was removed from the data, we dropped the left side landmarks for further analyses. Therefore, the effective number of landmarks for the analyses became 77 for the carapace and 25 for the plastron. From the symmetrised specimens, we computed a generalised Procrustes consensus configuration for each species. The code used to process the landmark data is given in Dataset S6.

In box turtles (*Cuora*), leaf turtles (*Cyclemys*), and *Notochelys*, the plastron is not always a single structure as it is usually divided along the transverse hinge that develops in these groups forming an anterior and a posterior plastral lobe. If there is a ‘natural resting angle’ between the two lobes, it would seldom be observed in osteological specimens. Instead of imposing an arbitrary angle between the lobes for the generalised Procrustes superimposition with the plastra of unhinged turtles, we artificially divided the unhinged plastra into anterior and posterior lobes. This way, generalised Procrustes superimpositions of anterior and posterior plastral lobes could be performed among all turtle species. Note that the division of the landmarks into anterior and posterior lobes only follows from this necessity and does not represent a priori hypotheses of modularity. Further, although those two parts are likely to become modules in hinged turtles for architectural and functional reasons, they are not necessarily true modules in species with unhinged plastra.

### Datasets

In order to assess the performance of individual superimpositions created in the previous step, and some of their combinations, we defined five datasets for the analyses (Table 3).

**Table 3:** Sets of landmark data defined for the phylogenetic analyses.

Dataset name	Content	Number of landmarks	Number of species sampled
*Carapace*	Carapace landmarks only	77	44
*Anterior lobe*	Anterior lobe landmarks only	14	46
*Posterior lobe*	Posterior lobe landmarks only	11	46
*Plastron*	*Anterior lobe + posterior lobe*	25	46
*Shell*	*Carapace + plastron*	102	42

For the sake of clarity, we use italics to refer to datasets. When we do not use italics, we refer to the anatomical structures after which the datasets are named. The species of the *shell* dataset correspond to the set intersection of the species of the *carapace* and *plastron* datasets. There is no missing data in any of the datasets.

The superposition steps were performed on the *anterior lobe*, *posterior lobe*, and *carapace* datasets independently. The *shell* and *plastron* datasets combine those independently superposed datasets, and therefore are not representations of the intact shell and plastron shapes. This is because, as noted in the previous section, it is not possible to produce superpositions of the entire plastra that include species that have mobile lobes, and for the same reason it is not possible to reconstruct the ‘correct’ position of the lobes relative to the carapace. Furthermore, some collection specimens of species with immobile plastra were prepared by sawing through the bridge and their carapace and plastra no longer fit back together perfectly, hindering the reconstruction the full shell shape in those cases as well.

### Phylogenetic analyses of morphometric data

We analysed our morphometric data using LAUP (spatial parsimony), SCP (Maddison, 1991; McArdle & Rodrigo, 1994; Rohlf, 2001), linear parsimony (Goloboff, Mattoni & Quinteros, 2006), neighbour-joining (Saitou & Nei, 1987) on Procrustes distances, and ML under a Brownian motion model of evolution (Felsenstein, 1973, 1981). For all methods we estimated a single optimal tree and performed a bootstrap analysis to quantify branch support. Bootstrap replications did not repeat the superposition step, only the tree searches. We took a 70% threshold as evidence of significant bootstrap support. This number is common in the discrete character literature, but arbitrary. To our knowledge, there are no studies analogous to Hillis & Bull (1993) that examine the properties of bootstrap support with continuous characters.

#### Selection of spatial optimisation parameters for LAUP

Landmark analysis under parsimony takes considerably longer time to run compared to traditional parsimony analysis because of the computationally-intensive heuristic spatial optimisation that must be performed to estimate ancestral landmark positions and compute the score of a tree topology. Roughly speaking, the standard optimisation strategy consists of applying a grid with a fixed number of subdivisions that discretises the space of possible positions for the ancestral landmark. After determining the optimal position of the landmark within that grid, further refinement can be done by repeating the process with a smaller grid centred around that position. Each refinement step is referred to as a ‘nesting’ level (Goloboff & Catalano, 2011). The number of grid divisions and grid-nesting iterations have been shown to have great impact on score estimation (Goloboff & Catalano, 2011), with a significant trade-off in computation time. In order to estimate reasonable grid subdivision and nesting parameters, we performed a series of trial analyses on the *shell* dataset with incremental values. We tried values of 6, 8, and 10 for the number of grid divisions, and values of 1 and 2 iterations of grid nesting with a cell window size of 1. These ranges of values were determined by our computational resources and are not atypical for the LAUP literature (Goloboff & Catalano, 2011; Catalano, Ercoli & Prevosti, 2015; Perrard, Lopez-Osorio & Carpenter, 2016). Combined, these are six parameter settings, for each of which we ran three LAUP searches of two random addition sequences followed by tree-bisection-and-reconnection (TBR) branch swapping. The outcome of the analyses was evaluated in terms of total computation time, tree score, and tree topology.

#### LAUP search strategies

We performed LAUP searches on all the datasets using GPA alignments and the dynamic alignment based on parsimony proposed by Catalano & Goloboff (2012). The heuristic search strategy for the GPA alignments consisted of 20 replicates of random addition sequences followed by TBR branch swapping using the default settings of TNT’s MULT command.

For the dynamic alignments, we used a search strategy conceived by Santiago Catalano (E. Ascarrunz, 2017, personal communication). It first performs a random pairwise alignment of the configurations followed by a tree search with a random addition sequence and a round of TBR. The tree obtained from that initial search is then fed into a loop of dynamic alignments followed by a tree search with TBR, until the same topology is obtained in two successive iterations. That whole procedure is performed eight times, yielding eight trees. Because eight is a very low number for random addition sequences with standard parsimony, but higher numbers were also prohibiting in computation time, we also performed an additional search using the reference tree as the starting tree instead. The tree with the lowest parsimony score of those nine searches is selected as the best estimate of the MPT.

Branch support was assessed using 100 replications of symmetric bootstrap resampling landmarks, each with one random addition sequence followed by TBR.

Searches with the GPA and dynamic alignments were performed with TNT 1.5 (Goloboff & Catalano, 2016).

#### Linear parsimony

We ran linear parsimony analyses that treat each coordinate of landmarks in a GPA superposition as an independent continuous character (Goloboff, Mattoni & Quinteros, 2006). The search strategy consisted of 5,000 random addition sequences followed by TBR branch swapping. Bootstrap was performed with 500 replicates, each analysed with 100 random addition sequences followed by TBR branch swapping.

#### Maximum likelihood analysis

Maximum likelihood analyses were performed directly on GPA superposition coordinates using CONTML v.3.696 from the Phylip package (Felsenstein, 2005). We did not attempt to correct for selective and genetic covariances between landmarks (Felsenstein, 1988, 2002).

The heuristic search settings for the ML tree consisted of 50 repetitions of a random addition sequence followed by tree improvement by subtree pruning and regrafting (SPR, or ‘global rearrangements’ in Phylip’s terminology). Bootstrapping was performed with 200 matrices resampling landmarks, each analysed with five repetitions of random addition sequence followed by SPR.

#### Neighbour-joining analysis

We constructed NJ trees from Procrustes distance matrices of each dataset using the R package ape (Paradis, Claude & Strimmer, 2004). For branch support assessment, we also computed NJ trees from 200 bootstrap replicates resampling landmarks.

#### Squared-change parsimony

To our knowledge, there is no published software capable of performing heuristic tree searches using SCP as an optimality criterion (but see Klingenberg & Gidaszewski, 2010 for a branch-and-bound implementation). Therefore, we wrote an R script for this purpose. The script computes the unweighted sum of squared changes on unrooted trees using the method described by Rohlf (2001; see also McArdle & Rodrigo, 1994; Claude, 2008). This implementation yields practically identical results to the anc.recon function for estimating ML ancestral states (Goolsby, 2017) included in the Rphylopars package (Goolsby, Bruggeman & Ané, 2017), when all branch lengths are set to unity (a punctuational model corresponding to unweighted SCP). This implementation is also slightly faster for datasets of the size used in this study. The code is written in pure R (Dataset S7), except for a single instruction that calls the C++ Eigen library (Bates & Eddelbuettel, 2013; Jacob, Guennebaud & Eigen Project Contributors, 2017) to perform matrix multiplication.

The search strategy consisted of performing 100 random addition sequences, each followed by hill-climbing optimisation using first random SPRs, and secondly with nearest-neighbour interchanges (NNI). The 25 trees with the best sum of squared changes scores were then used as starting trees for further optimisation with an implementation of the stochastic NNI perturbation search strategy of Nguyen et al. (2015), which was run 10 times. All the branch swapping operations made use of functions from the Phangorn package v.2.1.1 (Schliep, 2011). Bootstrapping was performed on 200 resampled matrices, each analysed with a single random addition sequence and the hill-climbing optimisation described above, but with less thorough settings.

### Topological accuracy and tree comparisons

All trees obtained from the morphometric analyses were compared against the reference tree in order to assess the accuracy of topological estimates. This assumes that the molecular data carries significant phylogenetic signal, and that it yields tree estimates that are better or at least as accurate as our morphometric data. The assumption can be empirically challenged if we find that the trees obtained from the different analyses of the landmark data are highly congruent and different from the molecular reference tree. Under such a scenario, it would be difficult to determine whether the congruence of the trees of different parts of the shell is either indicative of strong and reliable phylogenetic signal, or simply the result of the strong integration of the carapace and the plastral lobes.

Using a modified expression from Bogdanowicz, Giaro & Wróbel (2012), we define the topological accuracy (TA) of an estimated tree *T*_m_ with some dissimilarity measure as

$${\rm{T}}{{\rm{A}}_{\rm{d}}}\left( {{T_{\rm{m}}}} \right) = {{\grave{d}\left( {{T_{{\rm{ref}}}},{T_{{\rm{rand}}}}} \right) - \grave{d}\left( {{T_{{\rm{ref}}}},{T_{\rm{m}}}} \right)}\over{{{\grave{d}\left( {{T_{{\rm{ref}}}},{T_{{\rm{rand}}}}} \right)}}}},$$

where *d̀*(*T*_ref_, *T*_rand_) is the median dissimilarity between the reference tree and 10,000 random trees generated with the rmtree function of ape (for details on the generator algorithm, see Paradis, 2012: 313). Therefore, TA is 1 when there is no topological conflict between the observed tree and the reference tree, and is close to 0 when there is about as much topological conflict between the reference tree and the observed tree as between the reference tree and random trees on average.

The value of TA_d_ will, of course, depend on the dissimilarity measure used to compute it. The choice of dissimilarity measure is hardly ever simple, because each measure emphasises different topological features to the point that different measures can occasionally lead to different conclusions. For this reason, we compute TA_d_ with two dissimilarity measures. The first is the number of unrooted quartets that are resolved differently in the two trees (dQ) (the d quantity of Estabrook, McMorris & Meacham, 1985; also equivalent to an extreme case of the parametric quartet distance of Bansal, Dong & Fernández-Baca, 2011), which we computed with QDist v.2.0 (Nielsen et al., 2011). The other is the tree contradiction difference (CD) (Bapst, Schreiber & Carlson, 2018), defined as the number of incompatible splits between two unrooted topologies. We computed CD with the treeContradiction function of the paleotree package (Bapst, 2012). The TA computed with either measure is referred to as TA_dQ_ or TA_CD_, respectively.

The dissimilarity measures take only into account conflict between resolved relationships, ignoring differences corresponding to the presence of polytomies. This amounts to treating all polytomies as ‘soft’ in the sense of Maddison (1989). That is useful, because it allows us not to consider conflict with branches that are only poorly supported (with ultra-fast bootstrap support <95%) in the reference tree which we collapsed prior to the comparisons. However, because TA only takes into account resolved relationships, a fully unresolved tree (e.g. a majority-rule bootstrap consensus) is always going to be 100% accurate while also being fully uninformative. For this reason, we also report the amount of resolution of the trees, which we compute as the number of nodes that are present in the unrooted tree divided by *N* − 2, where *N* is the number of tips of the tree.

In addition to comparing the trees obtained from landmark data against the reference tree to assess error in topology estimation, we compared the landmark trees against each other to explore possible associations between topologies estimated from different datasets and different methods. For this, we computed pairwise distance matrices of all the trees using the quartet distance (Estabrook, McMorris & Meacham, 1985) and the Robinson–Foulds distance, which are closely related to the dQ and CD dissimilarity measures, respectively, but satisfy the conditions of true metrics (dQ and CD fail to satisfy the identity of indiscernibles and the triangle inequality for trees with polytomies). We used metric multidimensional scaling (MDS) (see Hillis, Heath & John, 2005 for a similar application) on the distance matrices to produce visualisations of the tree space projected on two dimensions and applied the partitioning around medoids algorithm (PAM) (Kaufman & Rousseeuw, 1990) in order to identify clusters of topologically similar trees. We determined the optimal number of clusters to be found by PAM, the *k* parameter, by means of the elbow method (Thorndike, 1953). In addition, we also applied PAM with *k* set to 5 and 6, to see if trees would cluster following the five datasets and/or the six methods of phylogenetic analysis.

Comparisons between trees estimated from landmark data required pruning out all the species not common to all datasets. After the pruning step, all the trees used for comparisons had 42 species.

### Fossil placement performance

To assess the potential performance of analyses of morphometric data for the placement of fossil species on a global reference phylogeny obtained by other means, we used a leave-one-out protocol derived from Garbin, Ascarrunz & Joyce (2018), which was in turn inspired by Berger & Stamatakis (2010). The procedure removes a species from the reference tree and reinserts it by estimating its position based on the morphometric data and some optimality criterion. This operation is performed on every species in the reference tree (except the outgroup), taken one by one. The quality of the placement of each species with the different methods is quantified by counting the number of nodes that separate the original (‘correct’) position in the reference tree and the point on which the species was reinserted. We scaled that quantity by the number of nodes separating the original position from the farthest possible reinsertion point.

We applied this protocol on the *carapace, plastron*, and *shell* datasets using GPA LAUP, ML, and SCP as optimality criteria. We did not use dynamic alignment with LAUP solely because of the excessive computational time required to realign the landmarks with every species on every possible placement position for the three datasets.

### Utility software

Computations were performed on a desktop computer using GNU Parallel build 20161222 (Tange, 2011) to make efficient use of all the CPU cores. A few utility functions in our scripts make use of the the phytools package v.0.6 (Revell, 2012). Tree plots were produced with FigTree v.1.4.2 (Rambaut, 2014) and the APE package v.5.1 (Paradis, Claude & Strimmer, 2004).

## Results

### Reference phylogeny

**Figure 2:**
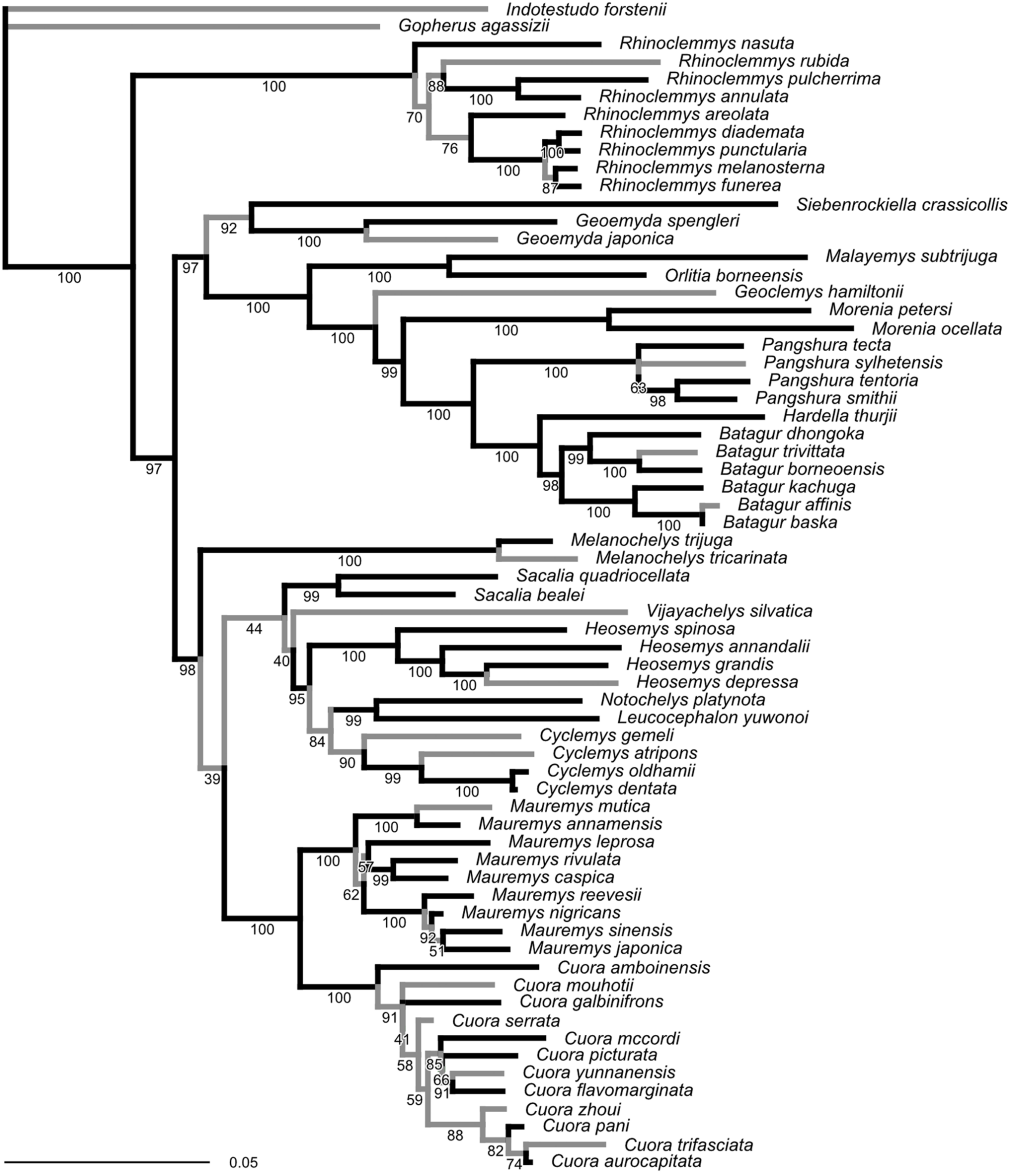
Phylogeny obtained from the ML analysis of seven molecular markers. Numbers under the branches are ultra-fast bootstrap values. The reference tree used to assess the performance of the analyses of landmark data was produced from this phylogeny by pruning or collapsing the branches in grey, and adding the emydid *Malaclemys terrapin* as the outgroup. The internal branches that were collapsed have less than 95% ultra-fast bootstrap support. The terminal branches that were pruned correspond to species for which we did not have landmark data. The scale is in expected number of nucleotide substitutions.

The ML tree obtained from our molecular sequence analysis (Fig. 2) is broadly concordant with recent global-level geoemydid phylogenies (Spinks et al., 2004; Diesmos et al., 2005; Guillon et al., 2012). All currently recognised ‘genera’ are recovered as monophyletic. Internal relationships of *Mauremys* and *Cuora* differ from the results of detailed analyses of these groups, but the different resolutions generally have low ultra-fast bootstrap support (<95%). *Rhinoclemmys* is recovered as sister to all other geoemydids (Spinks et al., 2004; Diesmos et al., 2005; contra Praschag et al., 2006; Sasaki et al., 2006) and *Geoemyda* and *Siebenrockiella* were found as sister to the *Orlitia–Batagur* clade (Spinks et al., 2004; contra Diesmos et al., 2005; Praschag et al., 2006). The latter point is also the only significant difference in the arrangement of major clades compared to the tree obtained from the molecular analysis of Garbin, Ascarrunz & Joyce (2018). This disagreement disappears once the bootstrap support for the clades in both trees is considered.

Unlike the traditional non-parametric bootstrap (Hillis & Bull, 1993), ultra-fast bootstrap support values have been found to be approximately unbiased estimates of the probability of a branch being correct (Minh, Nguyen & Von Haeseler, 2013). Therefore, we collapsed all branches with less than 95% ultra-fast bootstrap support for comparisons between the reference molecular phylogeny and the trees obtained from morphometric data, unless noted otherwise. The reference tree with unsupported branches collapsed is fairly conservative in relation to the results of recent molecular studies (Spinks et al., 2004; Diesmos et al., 2005; Praschag et al., 2006). Therefore, it seems unlikely that taking the collapsed reference tree as provisionally correct will be unduly severe for assessing the accuracy of the trees estimated from the morphometric data.

### Parameters for LAUP spatial optimisation

We identify a single topology as the most parsimonious in replications of four parameter combinations, including the most exhaustive one (10 grid divisions, two nesting iterations). We consider that topology to be the best estimate of the MPT for the GPA alignment of the *shell* dataset. Scaled quartet distances between the best tree and the trees obtained in other trials were never greater than 0.072.

As expected, parsimony scores improve with more thorough approximation settings (Table 4). Using two levels of grid nesting produced greater improvements in the tree score than increasing the number of grid subdivisions. The two-level nesting setting was also more effective in increasing the chance of finding the optimal topology.

**Table 4:** LAUP search trials with different landmark optimisation parameters.

Grid subdivisions	Nesting levels	Run	Score	Duration (hh:mm:ss)	Q to best tree
6	1	1	17.98179	00:53:41	0.010
6	1	2	17.99598	01:22:38	0.072
6	1	3	17.98170	00:58:15	0.010
**6**	**2**	**1**	**17.93101**	**01:36:26**	**0.000**
6	2	2	17.93784	01:41:11	0.065
**6**	**2**	**3**	**17.93096**	**01:42:58**	**0.000**
8	1	1	17.94410	04:55:02	0.004
8	1	2	17.96604	04:14:43	0.042
8	1	3	17.94413	04:12:55	0.004
**8**	**2**	**1**	**17.92453**	**07:43:48**	**0.000**
8	2	2	17.93497	07:51:28	0.065
**8**	**2**	**3**	**17.92448**	**05:54:17**	**0.000**
**10**	**1**	**1**	**17.93737**	**13:56:56**	**0.000**
10	1	2	17.95402	17:07:39	0.042
10	1	3	17.93807	14:58:16	0.021
**10**	**2**	**1**	**17.92531**	**26:45:34**	**0.000**
10	2	2	17.93228	22:33:37	0.065
**10**	**2**	**3**	**17.92531**	**21:52:13**	**0.000**

**Note:**

Three search runs were performed for each setting of grid divisions and nesting levels. The rows in bold indicate trials in which the most parsimonious topology was found. The last column is the scaled quartet distance (Q) to the best tree.

More exhaustive spatial optimisation settings slow down tree searches dramatically, raising search times from between 53 min and 1 h 30 min to over 26 h on mid-2014 MacBook Pro with a 2.2 GHz Intel Core i7 processor. For the following LAUP analyses in this study, we used six grid subdivisions and two grid nesting iterations, as searches with those parameters were able to find the best tree fast enough to allow for bootstrap analyses (search times of around 1 h 40 min).

### Phylogenetic analyses of morphometric data

For the optimality criterion methods (GPA and dynamic LAUP, linear parsimony, SCP, and ML) the tree scores obtained from the morphometric data analyses were always better than the scores of the reference tree (Table 5, trees available in Dataset S8). That suggests that any limited ability to recover relationships from morphometric data was not the result of suboptimal tree searches. This conclusion is also supported by the fact that we used the reference tree as the starting tree in LAUP, ensuring that the topological vicinity of the reference tree was explored. Searches initiated with the reference tree found better parsimony scores with dynamic LAUP three out of five times, whereas all the GPA LAUP searches initiated from random addition sequences outperformed the searches initiated from the reference tree.

**Table 5:** Accuracy relative to the reference tree and scores (parsimony or log-likelihood) of the optimal trees found.

Dataset	Method	Alignment	TA_dQ_	TA_CD_	Tree score	Reference tree score
*Anterior lobe*	LAUP	GPA	0.213	0.108	5.522	7.045
*Anterior lobe*	LAUP	Dynamic	0.225	0.162	5.269	6.586
*Anterior lobe*	SCP	GPA	0.202	0.135	0.070	0.116
*Anterior lobe*	LP	GPA	0.179	0.108	8.712	11.216
*Anterior lobe*	ML	GPA	0.195	0.135	8,153.209	7,833.224
*Anterior lobe*	NJ	GPA	0.194	0.162		
*Posterior lobe*	LAUP	GPA	0.232	0.135	5.377	6.708
*Posterior lobe*	LAUP	Dynamic	0.222	0.122	5.032	6.285
*Posterior lobe*	SCP	GPA	0.227	0.122	0.067	0.110
*Posterior lobe*	LP	GPA	0.219	0.162	7.510	9.443
*Posterior lobe*	ML	GPA	0.247	0.162	6,272.722	6,055.839
*Posterior lobe*	NJ	GPA	0.278	0.176		
*Plastron*	LAUP	GPA	0.221	0.135	11.278	13.753
*Plastron*	LAUP	Dynamic	0.212	0.162	10.649	12.872
*Plastron*	SCP	GPA	0.203	0.108	0.151	0.226
*Plastron*	LP	GPA	0.213	0.149	16.898	20.659
*Plastron*	ML	GPA	0.225	0.149	14,175.090	13,753.905
*Plastron*	NJ	GPA	0.247	0.162		
*Carapace*	LAUP	GPA	0.405	0.246	6.742	7.682
*Carapace*	LAUP	Dynamic	0.360	0.319	5.995	7.151
*Carapace*	SCP	GPA	0.324	0.290	0.032	0.043
*Carapace*	LP	GPA	0.263	0.232	15.397	17.845
*Carapace*	ML	GPA	0.317	0.246	54,196.470	53,287.233
*Carapace*	NJ	GPA	0.445	0.261		
*Shell*	LAUP	GPA	0.309	0.269	18.285	20.198
*Shell*	LAUP	Dynamic	0.319	0.269	16.340	18.856
*Shell*	SCP	GPA	0.249	0.179	0.191	0.246
*Shell*	LP	GPA	0.358	0.239	32.576	35.929
*Shell*	ML	GPA	0.284	0.254	59,202.235	58,325.959
*Shell*	NJ	GPA	0.283	0.239		

**Note:**

The reference tree scores for dynamic LAUP were computed by realigning the configuration in relation to the reference tree. Note that log-likelihood scores are positive.

Quantitatively, the overall performance of all methods with all datasets was poor (Table 5). The optimal trees obtained with each method had low accuracy, with TA_dQ_ values ranging in 0.179–0.445. Accuracy estimated with the CD (TA_CD_) was even lower, in the range of 0.108–0.319, in some cases about half the value of TA_dQ_. This large difference is likely due to the greater sensitivity of TA_CD_ to displacements of a few outlier species, an issue that also affects other split-based measures like the Robinson–Foulds distance (Böcker, Canzar & Klau, 2013). Nonetheless, TA_dQ_ and TA_CD_ broadly coincide on the accuracy of the trees relative to each other; the Pearson correlation coefficient between TA_dQ_ and TA_CD_ is 0.841.

**Figure 3:**
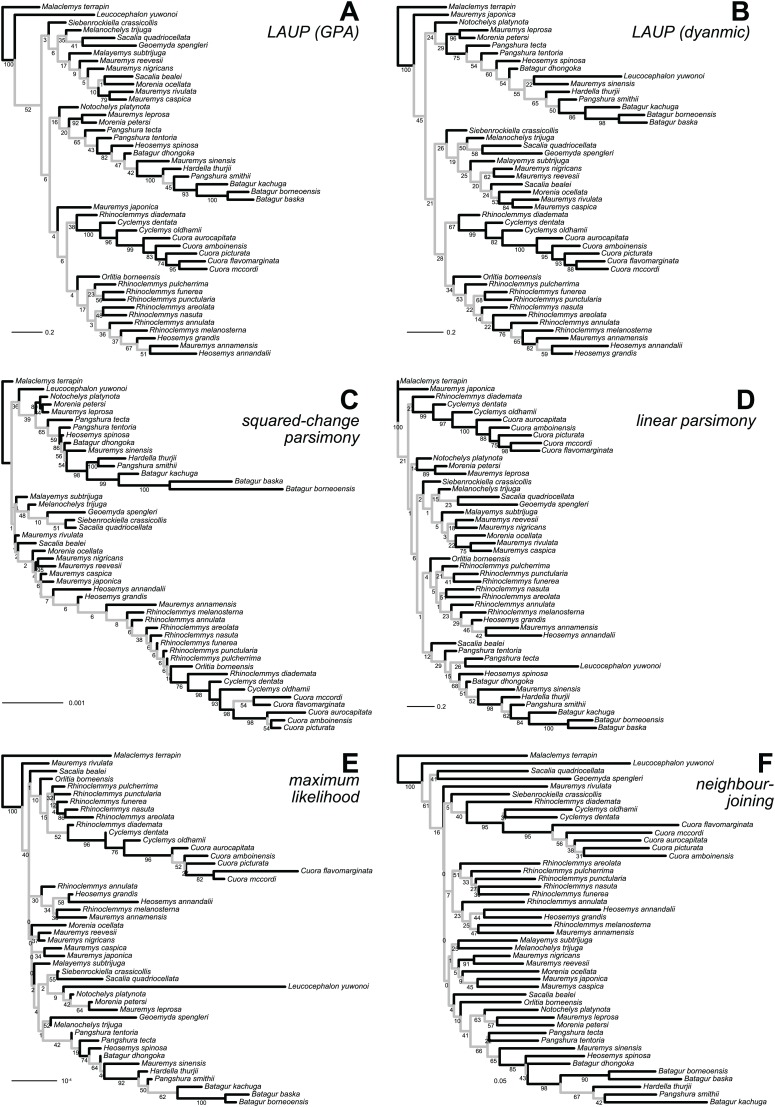
Optimal trees found for the *plastron* dataset with six inference methods. (A) LAUP of GPA-superimposed configurations. (B) LAUP with dynamic superimposition. (C) Squared-change parsimony. (D) Linear parsimony. (E) Maximum likelihood. (F) Neighbour-joining of Procrustes distances. The bootstrap support values are indicated under their respective branches; branches in light grey have bootstrap support <70%. Branch lengths are given in Euclidean distances of landmarks for LAUP and NJ, Manhattan distances for linear parsimony, squared Euclidean distances for square-change parsimony (SCP), and expected accumulated Brownian variance for maximum likelihood (ML).

**Figure 4:**
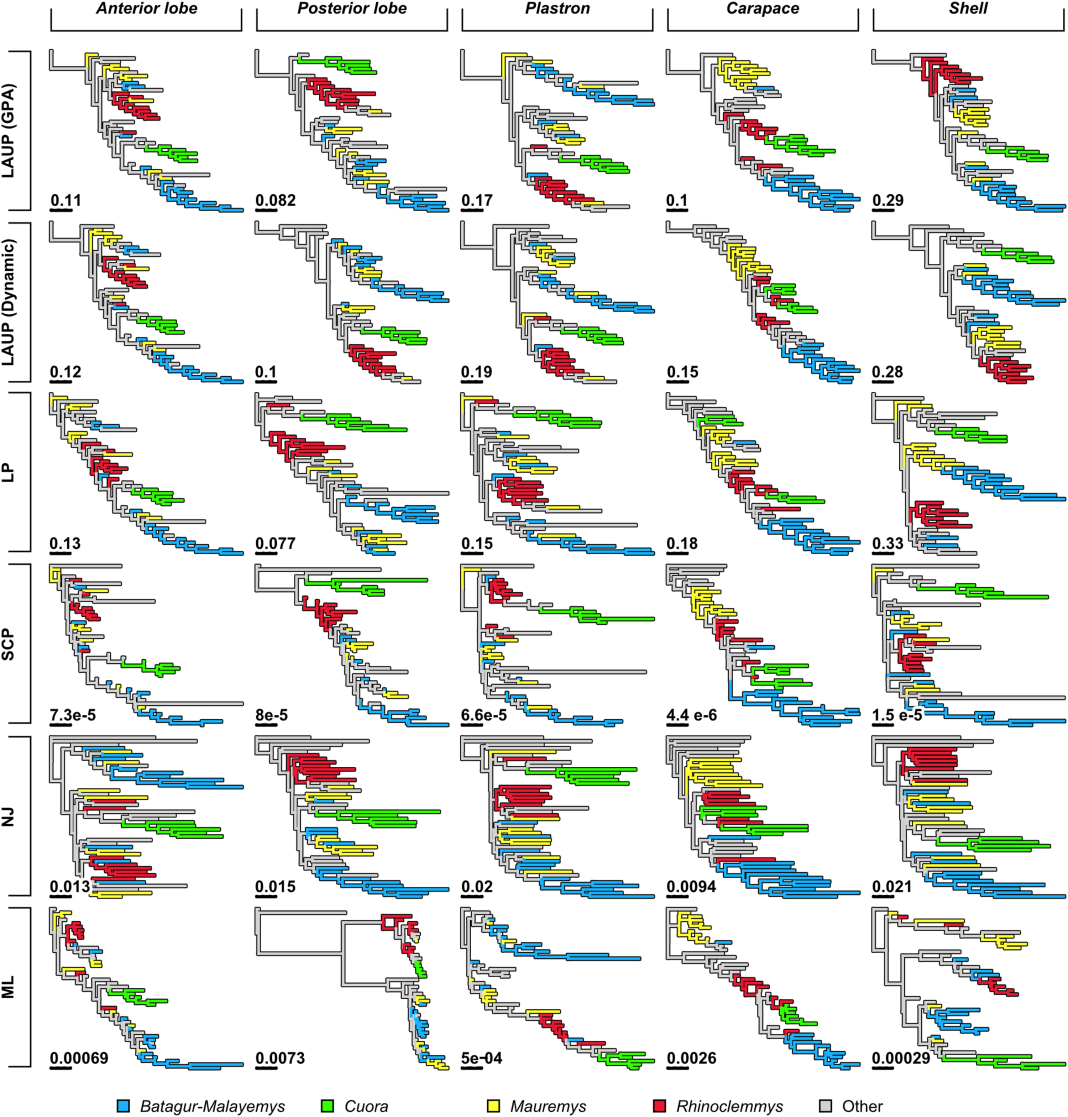
Trees found with the different methods and datasets, showing how closely together species of the clades Batagurinae, *Cuora*, *Mauremys*, and *Rhinoclemmys* were found. Branch lengths as in Fig. 3. Consult Fig. 3 and the Supplemental Material for the trees with all the species names.

**Figure 5:**
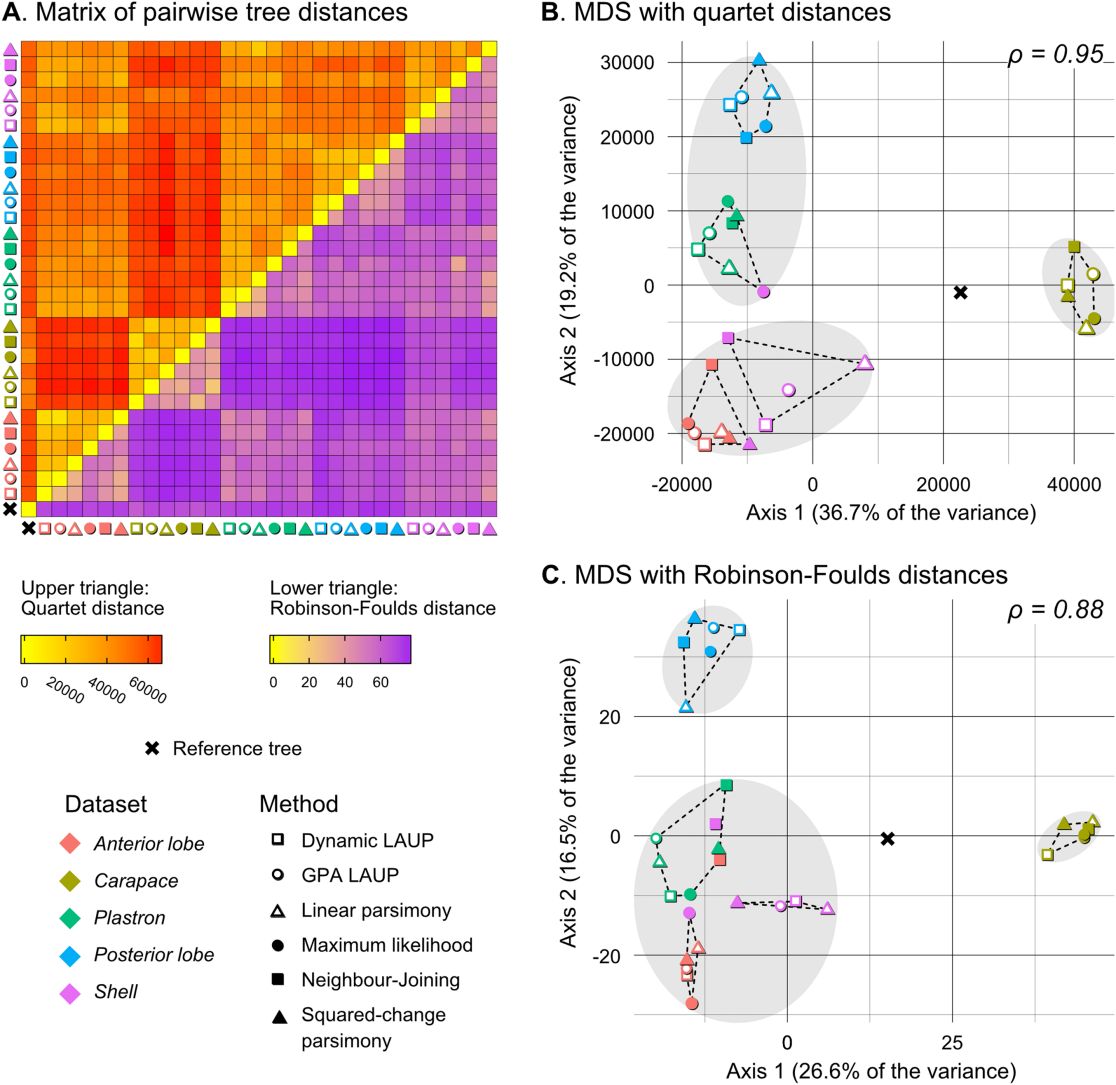
Quantitative comparison of tree obtained with different methods and datasets. (A) Matrix of pairwise distances, with the upper triangle showing quartet distances and the lower triangle Robinson–Foulds dsitances. (B) Plot of the first two axes obtained from metric multidimensional scaling (MDS) with quartet distances. (C) Plot of the first two axes obtained from metric multidimensional scaling (MDS) with Robinson–Foulds distances. The grey ellipses show the clusters identified with the PAM algorithm with *k* = 3, the optimum number of clusters according to the elbow criterion. The dashed lines show the convex hulls of clusters identified by PAM with *k* = 5, which is the number of datasets analysed. All trees were pruned down to their minimum set of species in common (*N* = 42). ρ is the correlation coefficient between the original tree distances and the distances in the MDS projection.

No analytical method or type of alignment showed a significant advantage or disadvantage over the rest. However, there were more consistent differences in performance between the different datasets (Table 5, see also Figs. 3–5). Analyses of the *anterior lobe* landmarks tended to perform the worst (median TA_dQ_ = 0.198, median TA_CD_ = 0.135), closely followed by the *posterior lobe* and *plastron* datasets with similar performance (median TA_dQ_ = 0.229 and median TA_CD_ = 0.149 for *posterior lobe*, and median TA_dQ_ = 0.217 and median TA_CD_ = 0.149 for *plastron*). The analyses of the *shell* and *carapace* datasets tended to be the most accurate (*carapace* median TA_dQ_ = 0.342, median TA_CD_ = 0.254; *shell* median TA_dQ_ = 0.296, median TA_CD_ = 0.2).

The 70%-rule consensus of bootstrap trees improved accuracy (Table 6). Predictably, this enhanced accuracy came at the expense of major losses of resolution; the most resolved consensus tree was obtained from the *carapace* dataset with neighbour-joining, with 52% of resolved nodes. Even that best-resolved tree is disappointing, as most of the resolved nodes only join pairs of species, failing to find major clades. The clades recovered more often in the 70%-rule bootstrap consensus included *Batagur*, *Pangshura*, and *Orlitia* (Fig. 3). We found no notable differences in total resolution or artefactual resolution (incorrectly resolved nodes) across the different methods.

**Table 6:** Topological accuracy and resolution of the 70%-rule bootstrap consensus of the analyses on morphometric data.

Configuration	Method	Alignment	TA_dQ_	TA_CD_	Internal nodes	Resolution
*Anterior lobe*	LAUP	GPA	0.952	0.784	8	0.182
*Anterior lobe*	LAUP	Dynamic	0.959	0.865	7	0.159
*Anterior lobe*	SCP	GPA	0.740	0.595	16	0.364
*Anterior lobe*	LP	GPA	0.953	0.811	8	0.182
*Anterior lobe*	ML	GPA	0.992	0.865	5	0.114
*Anterior lobe*	NJ	GPA	0.944	0.730	10	0.227
*Posterior lobe*	LAUP	GPA	0.945	0.730	8	0.182
*Posterior lobe*	LAUP	Dynamic	0.915	0.703	10	0.227
*Posterior lobe*	SCP	GPA	0.844	0.514	21	0.477
*Posterior lobe*	LP	GPA	0.981	0.797	8	0.182
*Posterior lobe*	ML	GPA	0.985	0.878	5	0.114
*Posterior lobe*	NJ	GPA	0.938	0.703	10	0.227
*Plastron*	LAUP	GPA	0.874	0.689	13	0.295
*Plastron*	LAUP	Dynamic	0.790	0.689	14	0.318
*Plastron*	SCP	GPA	0.818	0.568	16	0.364
*Plastron*	LP	GPA	0.946	0.743	12	0.273
*Plastron*	ML	GPA	0.882	0.730	9	0.205
*Plastron*	NJ	GPA	0.922	0.784	7	0.159
*Carapace*	LAUP	GPA	0.858	0.609	21	0.500
*Carapace*	LAUP	Dynamic	0.978	0.812	17	0.405
*Carapace*	SCP	GPA	0.967	0.783	16	0.381
*Carapace*	LP	GPA	0.922	0.667	18	0.429
*Carapace*	ML	GPA	0.919	0.681	16	0.381
*Carapace*	NJ	GPA	0.864	0.565	22	0.524
*Shell*	LAUP	GPA	0.942	0.731	16	0.400
*Shell*	LAUP	Dynamic	0.784	0.627	19	0.475
*Shell*	SCP	GPA	0.918	0.657	15	0.375
*Shell*	LP	GPA	0.932	0.687	18	0.450
*Shell*	ML	GPA	0.944	0.761	12	0.300
*Shell*	NJ	GPA	0.945	0.776	9	0.225

**Note:**

The resolution is given as the number of nodes observed in the tree divided by the number of nodes in a fully resolved tree with the same number of species. Trees were treated as unrooted.

Qualitatively, some trees present interesting features (Fig. 4). The reference tree has a large and well-supported clade of Asian turtles including *Batagur*, *Hardella*, *Pangshura*, *Morenia*, *Orlitia*, and *Malayemys* (Fig. 2). The trees from morphometric data often recover close relationships between the species in that *Batagur–Malayemys* clade, although some of those ‘genera’ are only occasionally recovered as monophyletic. The trees obtained from the *carapace* dataset tended to show all the *Mauremys* species clustering together either as a clade or as a paraphyletic assemblage, except for the linear parsimony tree. This is surprising, as *Mauremys* seems difficult to recover as a clade from its carapace alone (never recovered as monophyletic or paraphyletic in Garbin, Ascarrunz & Joyce, 2018), which does not seem very distinctive among geoemydids from our visual inspection of collection specimens.

The *anterior lobe*, *posterior lobe*, and *plastron* datasets yielded trees that consistently found *Cuora* as monophyletic, but with the two *Cyclemys* species often arranged as paraphyletic to *Cuora* (Figs. 3 and 4). This likely reflects the presence of a plastral hinge in these species. For instance, a close alignment of the pectoro–abdominal sulcus with the articulation between the hyoplastron and the hypoplastron is needed for a functional hinge, and is also present in species with partial plastral kinesis, such as *Heosemys spinosa* and *Vijayachelys sylvatica* (not observed in our sample, but reported in Moll, 1985, and references therein). The significant geometric signal produced by the presence of the hinge in testudinoids was demonstrated and studied in detail by Claude (2006). That study also showed that the hinge might be a source of confounding systematic characters, as it induces similar (albeit distinguishable) plastral morphologies in emydids and geoemydids. In our results, although the clustering of *Cuora* and *Cyclemys* clearly reflects an evolutionary convergence, it is encouraging that *Cuora* is always recovered as a clade, possibly because of features such as the rounded margins of its plastral lobes and the particular geometry of the entoplastral region, among others. The strength of that plastral signal is such, that all *shell* trees also recover *Cuora* as monophyletic.

It is noteworthy that many of the trees inferred from datasets that include plastral lobes also grouped together as a clade or paraphyletic assemblage all or almost all *Rhinoclemmys* species (Fig. 4), but this result is not as consistent across methods.

Quantitative comparison between trees is concordant with some of the previous observations (Fig. 5). The elbow method indicates that the tree spaces produced by quartet and Robinson–Foulds distances can be optimally partitioned into three clusters with the PAM algorithm (*k* = 3). In both tree spaces the greatest separation occurs along the MDS axis 1, between a compact cluster of trees derived from the *carapace* dataset and two clusters of trees derived from the *anterior lobe*, *posterior lobe*, *plastron*, and *shell* datasets. Among those two clusters, the trees inferred from the *anterior lobe* and *shell* datasets cluster together almost completely (the single exception is the ML *shell* tree in the quartet distance tree space). The trees inferred from the *plastron* dataset are found to cluster either with the *posterior lobe* trees (quartet distance tree space) or included in the cluster with the *shell* and *posterior lobe* trees (Robinson–Foulds distance tree space). These results are indicative of the strong influence of the plastral configurations, the anterior lobe in particular, in the overall signal detected by all methods. This influence must be due to specific geometric features, as the *carapace* dataset contains more than twice as many landmarks as the *plastron* dataset. The GPA superposition of the *anterior lobe* and *posterior lobe* have a between-species variance of 0.012 and 0.015, respectively, and the whole *plastron* has a variance of 0.013. In contrast, the *carapace* superposition has a variance of 0.002. Trees from the *shell* dataset still retain some of the better properties seen in the *carapace* trees, such as the better grouping of *Mauremys* and finding *Notochelys* close to *Cyclemys*.

We also applied PAM with *k* values of 5 and 6, to see if the trees would cluster reflecting the five datasets or the six methods of phylogenetic analysis used. We found five clusters that for the most part reflect the five landmark datasets. This corroborates that the datasets are the most important factor shaping the tree space even within the three main clusters indicated by the elbow method. PAM with *k* = 6 does not yield clusters that reflect the six phylogenetic methods.

### Fossil placement performance

**Figure 6:**
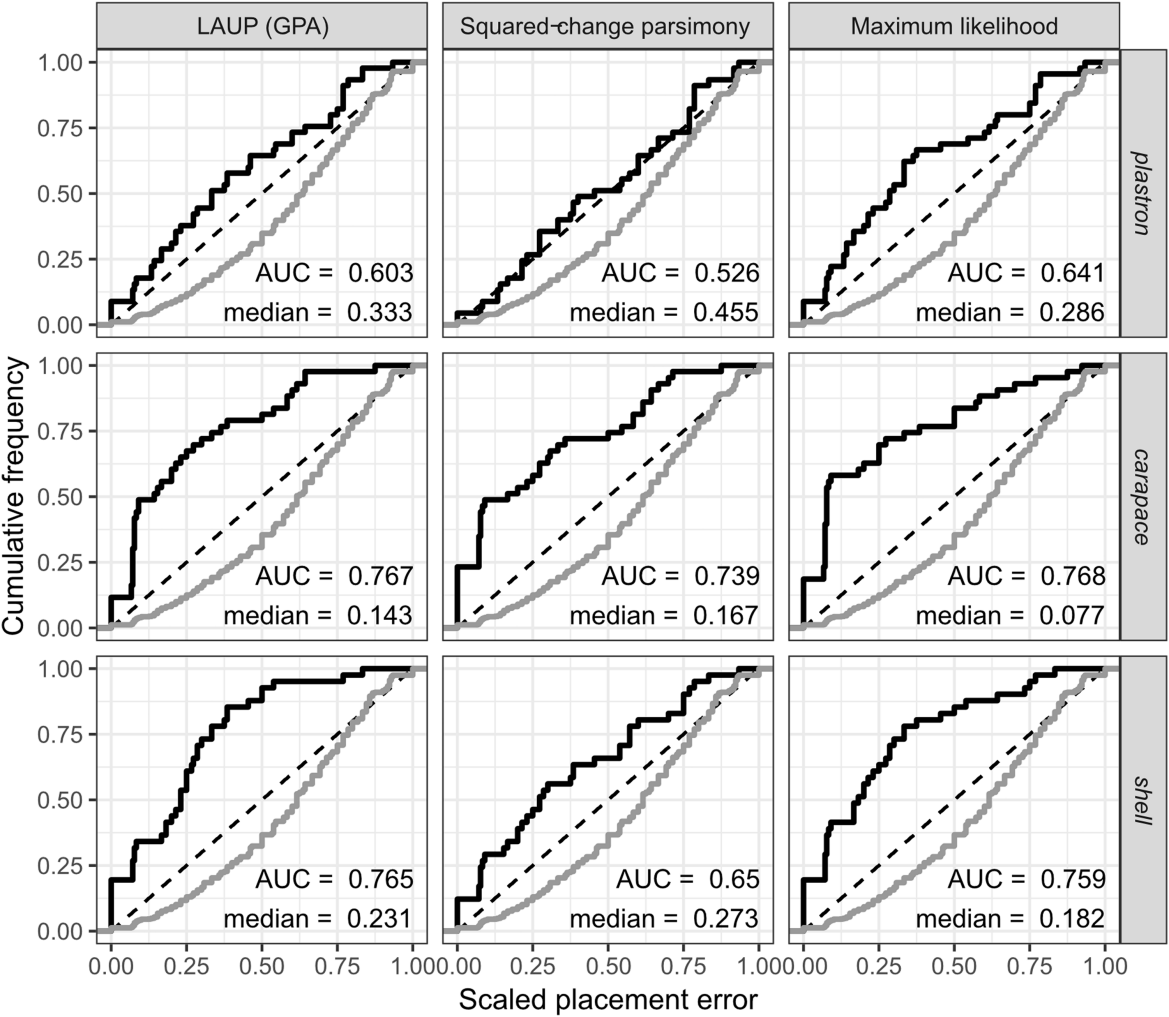
Cumulative frequency distributions of the placement error in fossil placement analyses (in black), measured by scaled nodal distances. Greater areas under the curve (AUC) and smaller medians reflect greater proportions of nodes with little placement error. The cumulative frequency distributions of the scaled nodal distances of all the possible palcements are shown in grey.

The fossil placement analyses consist in removing a single species from the reference tree and inserting it back into the tree based on the landmark data. This is done for all species in the tree, one by one. The results show some differences in performance across datasets, with the *plastron* dataset yielding the greatest scaled nodal errors, and *carapace* the least (Fig. 6). Additionally, ML performed consistently better than GPA LAUP and SCP, as measured by the median and the integrated cumulative distribution of the median scaled error. The differences are small and appear most pronounced for the *carapace* dataset, where the median scaled placement error with ML (0.077) was less than half the median error with GPA LAUP and SCP (both 0.167). For the *carapace* dataset, scaled placement errors in the range 0.067–0.09 correspond to placements at only one node away from the original position in the reference tree. With ML, the 70th percentile of the scaled placement errors reflects up to three nodes of separation between the reinsertion point and the original (‘correct’) position on the reference tree.

The fossil placement analyses were performed with the fully resolved reference tree that includes many branches that are poorly supported by molecular data (Fig. 2). Therefore, at least a small amount of the placement error can be reasonably ascribed to the incorrect branching patterns in the reference tree. Considering this, the performance of fossil placement analyses is much better than the performance of full phylogenetic analyses, at least for the *carapace* and *shell* datasets.

## Discussion

### Performance of methods of phylogenetic analysis

No method consistently yielded more accurate and precise trees. In this respect, our results are in agreement with the study of Catalano & Torres (2017), who compiled 41 landmark datasets and analysed them with five phylogenetic methods including neighbour-joining of Procrustes distances, linear parsimony, and LAUP (GPA). They found little difference in the performance of all methods. Their results and ours suggest that the current methods of phylogenetic inference and landmark datasets available do not directly address the major obstacles in making reliable phylogenetic inferences from geometric information. In our case the results suggest that the poor performance is particularly driven by low phylogenetic information and/or high noise (convergence) in the data, and the differences between trees from different methods are attributable to comparatively minor effects of the search strategies and optimality criteria. Indeed, the topological vicinities demonstrated in Fig. 5 show that there is some significant degree of agreement between the methods, and a Shimodaira–Hasegawa test (Shimodaira & Hasegawa, 1999) of all the trees obtained from the *anterior lobe* dataset shows that none of the trees yielded by the other methods has a significantly worse likelihood than the ML tree (all *p*-values > 0.26, test performed with Phylip’s CONTML). In contrast, the molecular reference tree has a significantly lower likelihood with the *anterior lobe* data (*p* < 0.001). We take this to be indicative of all the methods being misled and converging into the same optimality plateau, away from the more plausible molecular tree. Previous studies have found evidence of significant convergence of shell shapes in testudinids (Claude et al., 2003; Claude, 2006), and this is likely part of the signal that is misleading all the methods.

These problems apply even to LAUP, which was specifically conceived for a proper treatment of landmark characters consistent with the general framework of maximum parsimony. Our heuristic searches under LAUP were severely limited by the computational demands of the numerical approximation algorithms involved. However, we should note that we also gave LAUP an additional ‘advantage’ by launching searches using the reference tree as the starting point. Furthermore, the searches performed by Catalano & Torres (2017) were more exhaustive than ours, and their overall findings are the same. Other two major studies making use of LAUP have been Perrard, Lopez-Osorio & Carpenter (2016) and Jones & Butler (2018). Perrard, Lopez-Osorio & Carpenter (2016) used dynamic LAUP of wing venation patterns in landmark-only and total-evidence analyses of Vespinae. They found that landmarks alone would not yield reliable results, and the addition of landmark characters to their molecular and discrete character data affected only poorly supported nodes and was reasonably attributable to noise. Jones & Butler (2018) conducted phylogenetic analyses of phytosaurs with different versions of a matrix with mostly discrete characters and subsets of characters represented in either discrete, continuous, or landmark form, or a combination of continuous and landmark form. Compared to the present study, their analyses ran more thorough heuristic searches and made use of implied weighting (Goloboff, 1993, 2014), but did not perform the computationally expensive dynamic realignment of 3D configurations (all their landmark characters were two-dimensional and the configurations were aligned by GPA). They found that the inclusion of landmark characters had a small topological effect compared to univariate continuous characters, and was accompanied by reduction in nodal support. The authors favoured the trees obtained from discrete characters alone and discrete combined with continuous characters.

Squared-change parsimony has long been recommended for the treatment of morphometric shape variables in a phylogenetic context (Rohlf, 2001, 2002; also criticised by Catalano, Goloboff & Giannini, 2010), but its use for phylogenetic inference has remained limited to small datasets that do not require heuristic searches (Klingenberg & Gidaszewski, 2010). Here, we presented the first implementation, to our knowledge, of heuristic searches with the minimisation of the sum of squared changes as the optimality criterion. In our study, SCP often performed worse than other methods with our data (except with the *carapace* dataset), but the performance of all the methods was poor, with TA_dQ_ values typically lower to 0.4. Further studies will be needed to arrive at more general conclusions about the performance and utility of SCP in phylogenetic analysis using continuous characters. As expected with any kind of data analysed with methods that perform similarly, the greatest impact on topology was determined by the particular properties of the samples chosen. The datasets yielded recognisably different sets of topologies, although none consistently better than the others in terms of accuracy, and in the fossil placement analyses the *carapace* dataset outperformed the *shell* and *plastron* datasets. At the same time, the number of landmarks in the configurations was not the major factor driving the topology when multiple configurations were included in the same analysis. This highlights the importance of configuration selection in analyses in general. Catalano, Ercoli & Prevosti (2015) performed LAUP analyses of mustelids based on nine configurations from diverse osteological elements and found that the topology inferred from landmarks converged with their molecular reference tree as more configurations were included. As our configurations are not as numerous and feature degrees of functional and geometric integration (the shell as a character complex), our study should not be taken as strong evidence against a general rule of thumb that increasing the number of independent configurations sampled improves tree inference from landmark data, but serves as a reminder to be mindful of the influences of individual configurations, especially when the total number of samples is low.

Further studies would be needed to evaluate whether atomising the shell into modules would be helpful for identifying independently evolving landmark sets and improving phylogenetic accuracy. Even if a priori selection of modules could be tempting, it should be kept in mind that the delimitation of developmental modules can itself evolve, making this kind of approach complicated in practice. For instance, in geoemydids, it is clear that the acquisition of a hinge between plastral lobes will generate functional and architectural constraints that would likely alter module delimitations between clades that have mobile plastra and those who don’t.

Given all the above, we have no grounds in performance to recommend any of the methods we studied over the rest, but other practical considerations are still of interest. The Brownian motion model that we used here under the criterion of ML is also naturally applicable to Bayesian inference (Parins-Fukuchi, 2017, 2018), and the field of Bayesian analysis of morphological characters is developing rapidly (see Wright, 2019 for an overview of the state of the art). Much work remains to be done, but the software RevBayes (Höhna et al., 2016) has emerged as a flexible and performant platform for Bayesian phylogenetic analysis, and seems suitable for future experimentation with continuous characters and their integration with more traditional discrete morphological characters and molecular data. On the parsimony side, similar tools are available on TNT and already mature, despite the recency of LAUP. However, the computational burden of LAUP might discourage potential users, especially since in the studies published to date landmark data has not decisively altered the conclusions reached from discrete data. From a more positive angle, our results suggest that simple linear parsimony performs about as well as LAUP with empirical data, and therefore parsimony users could take advantage of linear parsimony at the very least for kick starting LAUP searches.

### Prospects on the utility of morphometric data

Our results show that phylogenetic analyses of morphometric shell data can recover close relationships of species in clades validated by molecular data, particularly some of the ‘genera’ in our ingroup. However, most of those relationships receive poor bootstrap support, and more global relationships inferred from morphometric data are much more inconsistent between changes of analytical method and bear little resemblance to our molecular reference tree. The failure of morphometric data to recover deep nodes is comparable to the performance of discrete morphological shell characters (Garbin, Ascarrunz & Joyce, 2018), and shows that the phylogenetic signal of morphology with either approach is too weak to suffice for the inference of the global phylogeny of geoemydids with an acceptable level of confidence.

The set of structures represented by our morphometric data does not completely overlap with the set of structures represented in the discrete morphological matrices. The morphometric representation fails to consider classic diagnostic characters, such as the number and position of longitudinal carapacial keels and musk duct foramina. Conversely, a recent revision of discrete shell characters (Garbin, Ascarrunz & Joyce, 2018) does not include characters for the overall shape of the carapace, and very few characters pertaining the angles formed by the bone sutures and the scute sulci. Furthermore, from the results of the present study, it is apparent that the morphometric data can capture synapomorphies not previously found using discrete character coding. These potential morphometric synapomorphies allowed the methods to frequently recover most or all the *Mauremys* species as a clade in the *carapace* trees and almost all the *Rhinoclemmys* clustering together as paraphyletic in the *plastron* trees. Those two clades are difficult to recover with discrete morphological characters.

Given the limitations of the landmark data of the present study and the discrete matrix from Garbin, Ascarrunz & Joyce (2018), it becomes clear that inferences about the phylogenetic position of fossil geoemydids will need to rely on molecular data to resolve the global relationships within the clade.

**Figure 7:**
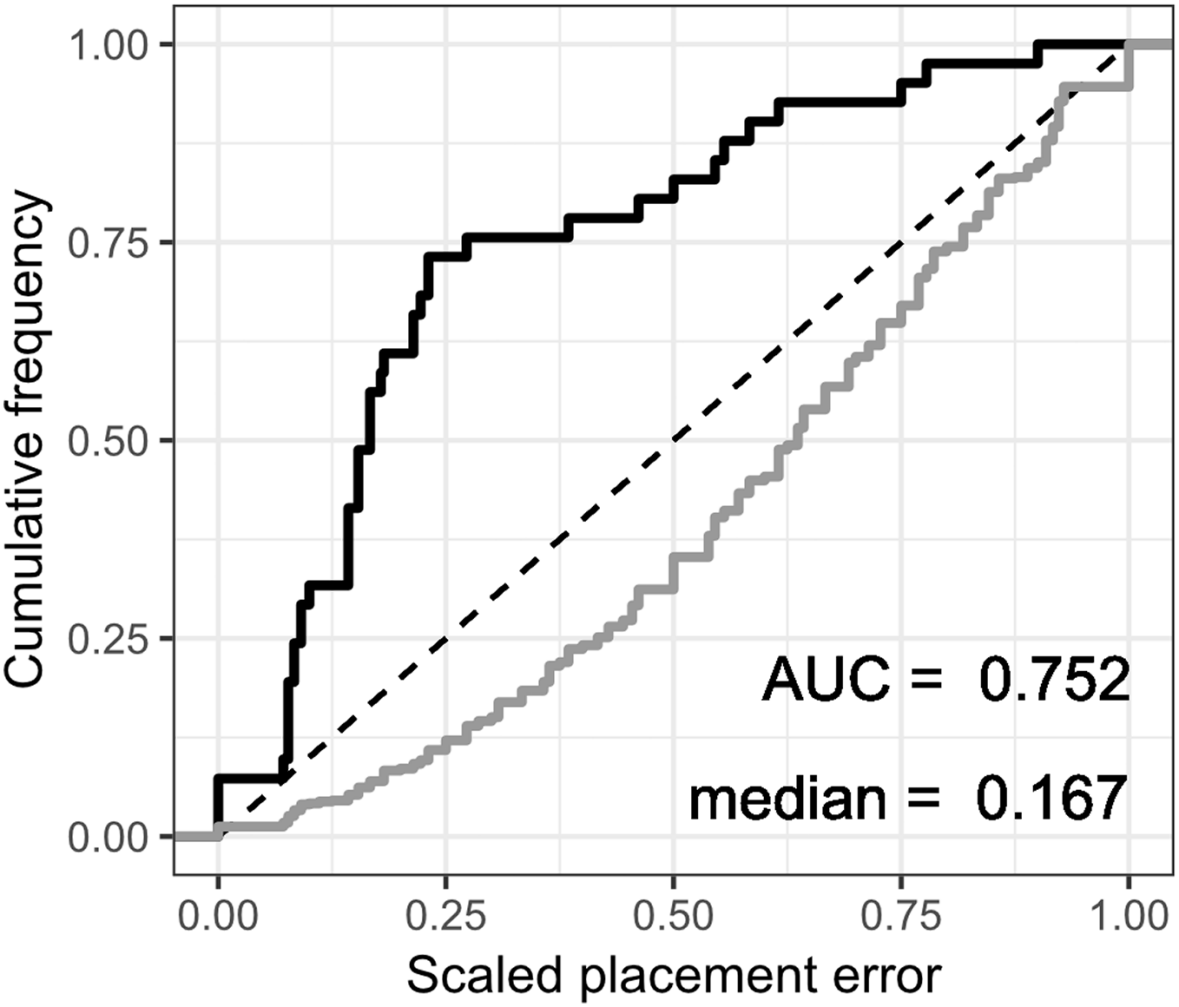
Placement error in fossil placement analyses of the discrete characters. Maximum parsimony fossil placement analyses of the character matrix from Garbin, Ascarrunz & Joyce (2018). The figure and the analyses are new, and they include the 39 species that are also present in the reference tree of this study. The median scaled nodal distance was used when multiple most parsimonious placements were found for a single species. Please also note that there is no similar figure in Garbin, Ascarrunz & Joyce (2018).

A way to achieve the integration of morphological and molecular data is by means of fossil placement analyses on a molecular scaffold. We evaluated the performance of those analyses with landmark data in this study, and Garbin, Ascarrunz & Joyce (2018) did the same with discrete characters. Globally, the accuracy of the discrete characters from the entire shell is very similar to the accuracy obtained by *carapace* landmarks alone (Figs. 6 and 7), with the landmark data also having the advantage of giving fully resolved relationships whereas the discrete data yielded multiple optimal parsimony placements for 56% of the species (number of most parsimonious placements: median 3, maximum 15). The performance of landmarks from the entire *shell* dataset was slightly worse. The fact that the mere accumulation of the configurations (i.e., increasing the number of shape variables) does not improve phylogenetic inference from landmarks in our data highlights the importance of misleading signal from the *plastron* data, which is particularly unfortunate, as this is the structure whose geometry is best preserved in fossils, and the necessity to boost the signal of the most reliable characters. Parins-Fukuchi (2018; based on previous work by Berger & Stamatakis, 2010) presented a method for continuous data that calibrates characters (e.g. landmarks) according to their fit to a reliable phylogeny obtained by external means (e.g. molecular analyses). We will explore this approach in an upcoming contribution.

From a practical perspective, geoemydid shells are almost never found with their intact original geometry, which hampers the utilisation the *carapace* landmarks. However, a strength of landmarks as a means of data acquisition and encoding is that a wealth of linear characters can be derived from them, and therefore it should be possible to identify linear measurements corresponding to localised shell features that would not be strongly affected by global deformation. Among such measurements, valuable phylogenetic characters may be discovered. Our future work will present a simple method to achieve this.

## Conclusions

Our results show that geoemydid shell landmark data behave similarly to traditional discrete shell characters: they do not suffice for the reliable estimation of phylogenetic relationships, regardless of the method of analysis used, but their combination with molecular trees in phylogenetic placement analyses yields more promising results. More work will be needed to make practical the application of landmark data in palaeontological studies of geoemydids, particularly because the structures that bear the strongest phylogenetic signal are not the ones best preserved in fossils. We believe that such further effort is warranted, nevertheless, as our landmark datasets and the currently available discrete character matrices do not cover completely overlapping sets of morphological features, and as much observed variation is better represented by continuous measurements anyway.

## Supplemental Information

10.7717/peerj.7476/supp-1 Supplemental Information 1 Summary of sampled specimens. Includes collection number, identification down to the species or subspecies level, sex (when available). The filled circles indicate when the carapace and the plastron were sampled. All the specimens are considered adults according to collection labels, size, and well-known developmental traits. Collection abbreviations: FMNH, Field Museum of Natural History; MCZR, Museum of Comparative Zoology Reptile Collection; PCHP, Chelonian Research Institute; YPM, Yale Peabody Museum.

10.7717/peerj.7476/supp-2 Supplemental Information 2 Number of sampled specimens per species. Detail of how many carapaces and plastra were sampled per species to build the datasets used in the phylogenetic analyses.

10.7717/peerj.7476/supp-3 Supplemental Information 3 DNA data for the construction of the reference tree. Contains the alignment of multiple DNA sequences and the NEXUS file with the partition and model specifications used for the estimation of the reference tree.

10.7717/peerj.7476/supp-4 Supplemental Information 4 Complete list and metadata of the specimens examined. The column ‘lisu’ contains the accepted systematic identification of the specimen as used in all the analyses. Institutional abbreviations as in Table S1.

10.7717/peerj.7476/supp-5 Supplemental Information 5 Definitions of the carapace landmarks.

10.7717/peerj.7476/supp-6 Supplemental Information 6 Definitions of the plastron landmarks.

10.7717/peerj.7476/supp-7 Supplemental Information 7 The raw landmark data from collection specimens in TPS format. Each specimen is referred to by its collection number, and replicate measurements of the same specimen are indicated by the character ‘$’ followed by the letter ‘a’ or ‘b’.

10.7717/peerj.7476/supp-8 Supplemental Information 8 R scripts and configuration files for processing the raw landmark data in the ‘carapace.tps’ and ‘plastron.tps’ files. The scripts ‘carapace_average.R’ and ‘plastron_average.R’ produce TPS files with Procrustes-superimposed species configurations of the carapace, anterior lobe, and posterior lobe. Carapace abnormalities are corrected in 10 specimens by replacing the landmarks in the ‘dont_use’ column in the ‘abn_fix.csv’ file with their reflected lateral counterparts in the ‘use’ column in the same file.
The file ‘utility_functions.R’ contains various functions that are called by the other scripts, as well as the functions ‘writecont.tnt’ and ‘writeland.tnt’, which can be used for producing TNT data files for linear parsimony and LAUP analyses.

10.7717/peerj.7476/supp-9 Supplemental Information 9 R scripts used for squared-change parsimony analyses. ‘routines.R’ contains the functions for computing squared-change parsimony scores and conducting heuristic searches with the squared-change parsimony criterion. ‘mprod.cpp’ contains minimal C++ code that is used for calling the Eigen library in ‘routines.R’. Searches from random addition sequences were done with the script ‘ras.R’. Searches with stochastic NNI perturbations were done with the script ‘stochastic.R’ Bootstrap searches were done with ‘bootstrap.R’.

10.7717/peerj.7476/supp-10 Supplemental Information 10 The optimal trees from all the analyses in Newick format. The trees are organised in folders corresponding to each landmark dataset. Within each folder the trees are named as follows:
‘dyn’ for dynamic LAUP
‘gpa’ for LAUP on GPA alignment
‘lin’ for linear parsimony
‘ml’ for maximum likelihood
‘nj’ for neighbour-joining
‘sqcpars’ for squared-change parsimony
‘_bs’ and ‘_bscon’ denote bootstrap trees, and bootstrap consensus trees, respectively.

## Additional Information and Declarations

### Data Availability

The following information was supplied regarding data availability: The raw data and code are available in the Supplemental Files. The raw landmark coordinates are available in Dataset S5, and the collection data of the corresponding specimens is given in Dataset S1. The specimen accession numbers for all 175 specimens studied are available in Dataset S2. The biological definitions of the landmarks are provided in Datasets S3 and S4. Dataset S6 contains the scripts used for producing cleaning up the data and producing the landmark datasets for the phylogenetic analyses. Dataset S7 provides the custom R code for the squared-change parsimony analyses. The trees obtained from the phylognetic analyses of landmark data are given in Dataset S8.
